# Genetic Background of *Escherichia coli* and Extended-spectrum β-Lactamase Type

**DOI:** 10.3201/eid1101.040257

**Published:** 2005-01

**Authors:** Catherine Branger, Oana Zamfir, Sabine Geoffroy, Geneviève Laurans, Guillaume Arlet, Hoang Vu Thien, Stéphanie Gouriou, Bertrand Picard, Erick Denamur

**Affiliations:** * Hôpital Louis Mourier, AP-HP, Colombes, France;; † INSERM E0339, Paris, France;; ‡ Hôpital Nord, Amiens, France;; § Hôpital Tenon, Paris, France;; ¶ Hôpital Trousseau, Paris, France;; # Hôpital Morvan, Brest, France; * Hôpital Louis Mourier, AP-HP, Colombes, France; † INSERM E0339, Paris, France; ‡ Hôpital Nord, Amiens, France; § Hôpital Tenon, Paris, France;; ¶ Hôpital Trousseau, Paris, France; # Hôpital Morvan, Brest, France

**Keywords:** Escherichia coli, extended-spectrum β-lactamase, virulence, phylogenetic group, fluoroquinolone resistance, research

## Abstract

To assess the implication of the genetic background of *Escherichia coli* strains in the emergence of extended-spectrum-β-lactamases (ESBL), 55 TEM-, 52 CTX-M-, and 22 SHV-type ESBL-producing clinical isolates involved in various extraintestinal infections or colonization were studied in terms of phylogenetic group, virulence factor (VF) content (*pap*, *sfa*/*foc*, *hly*, and *aer* genes), and fluoroquinolone resistance. A factorial analysis of correspondence showed that SHV type, and to a lesser extent TEM type, were preferentially observed in B2 phylogenetic group strains that exhibited numerous VFs but were fluoroquinolone-susceptible, whereas the newly emerged CTX-M type was associated with the D phylogenetic group strains that lacked VF but were fluoroquinolone-resistant. Thus, the emergence of ESBL-producing *E. coli* seems to be the result of complex interactions between the type of ESBL, genetic background of the strain, and selective pressures in ecologic niches.

## Abstract 2

ESBL-producing *E. coli* may arise from interactions between ESBL type, strain genetic background, and selective pressures in various ecologic niches.

Extended-spectrum β-lactamases (ESBL) that mediate resistance to oxyimino-cephalosporins, such as cefotaxime, aztreonam, and ceftazidime, are now observed worldwide in all species of *Enterobacteriaceae* (*1*). Traditionally, ESBLs are derived by point mutation from the common TEM and SHV-1 β-lactamases. However, recently, new families of ESBLs have been described (*2*). The CTX-M-type ESBLs have become particularly widespread and are mainly found in strains of *Salmonella* and *Escherichia coli* (*3*,*4*). These enzymes probably evolved from chromosomal β-lactamases of *Kluyvera* spp. by gene transposition from mobile elements and mutation (*5*,*6*). ESBLs are usually described as acquired β-lactamases that are encoded mainly by genes located on plasmids. Some ESBL-encoding genes are located within transposons or integrons, which facilitates transfer between organisms. ESBL-producing organisms are responsible for nosocomial infections, and many hospitals have experienced outbreaks (*1*,*2*,*7*). The lower digestive tract of colonized patients has been recognized as the major source of ESBL-producing organism (*2*,*8*). These organisms pose a therapeutic challenge, since they are frequently resistant to other kinds of antimicrobial drugs, including aminoglycosides, quinolones, and cotrimoxazole (*2*).

*E. coli* in humans is a commensal inhabitant of the gastrointestinal tract. It can also cause various intestinal and extraintestinal diseases (*9*). Strains causing infections harbor numerous virulence factors encoded on plasmids, bacteriophages, or the bacterial chromosome within pathogenicity islands (*9*). Several studies have shown that pathogenic *E. coli* strains may be derived from commensal strains by acquiring chromosomal or extrachromosomal virulence operons (*10*,*11*). Phylogenetic analyses have shown that *E. coli* strains fall into 4 main phylogenetic groups (A, B1, B2, and D) (*12*,*13*). Although virulence determinants are considered to be mobile, a link between strain phylogeny and virulence has been reported. Virulent extraintestinal strains belong mainly to group B2 and, to a lesser extent, to group D, whereas most commensal strains belong to groups A and B1. Strains of phylogenetic groups B2 and D often carry virulence determinants that are lacking in group A and B1 strains (*10*,*14*–*17*). In addition, a trade-off between resistance and virulence has been observed. Prevalence of antimicrobial resistance was shown to be greater in non-B2 phylogenetic group strains (*18*). In urinary tract infections, fluoroquinolone-resistant *E. coli* represented predominantly low-virulence phylogenetic groups A and B1 (*19*). These resistant strains were also associated with a decrease in the presence or the expression of some virulence factors and a decreased invasive capacity (*20*,*21*).

The intrinsic virulence potential of ESBL-producing *E. coli* is unknown. They may represent traditional virulence clones of extraintestinal pathogenic *E. coli* (ExPEC) or low-virulence opportunists whose ability to cause disease is largely limited to compromised hosts, in which antimicrobial resistance might provide relevant selective advantage. To assess the relationships between the genetic background of the strains and the presence of an ESBL, we analyzed a collection of ESBL-producing *E. coli* clinical isolates involved in various extraintestinal infections or in colonization in terms of phylogenetic grouping, virulence determinant content, and fluoroquinolone resistance.

## Material and Methods

### Bacterial Strains

We collected 157 *E. coli* isolates from clinical samples on the basis of their positive double-disk synergy test from 1997 to 2002 in different areas in France: Paris area (4 hospitals), Brest, and Amiens. From these isolates 129 strains were analyzed on the basis of 3 criteria: 1) the strains produced an ESBL, 2) the strains were epidemiologically unrelated, and 3) the strains were unambiguously classified as responsible for infection or colonization. ESBLs were characterized by isoelectric focusing with ceftriaxone and penicillin as substrates (*7*), specific polymerase chain reaction (PCR) amplification, and direct sequencing of PCR products. The oligonucleotide primer sets specific for the β-lactamase gene (*bla*) amplification and sequencing were taken from the literature (*bla*_TEM_ and *bla*_SHV_) (*22*) or designed in this study (*bla*_CTX-M_) (Table 1). As the family of CTX-M ESBLs belongs to 4 clusters on the basis of their protein sequences, the CTX-M-1 cluster (CTX-M-1, CTX-M-3, CTX-M-10, CTX-M-12, CTX-M-15), the CTX-M-2 cluster (CTX-M-2, CTX-M-4 to CTX-M-7, Toho-1), the CTX-M-9 cluster (CTX-M-9, CTX-M-14, CTX-M-16, CTX-M-18, CTX-M-19, Toho-2), and the CTX-M-8 cluster, specific primers for each cluster of the CTX-M family were designed. PCR products of *bla*_TEM_ were subjected to direct sequencing to identify TEM-ESBLs, only when isolates produced a single β-lactamase indicated by isoelectric focusing. For isolates carrying a second β-lactamase of pI 5.4 or 5.6 shown by penicillin only (putative TEM-1 or TEM-2 β-lactamase), sequences were obtained after plasmid transfer into *E. coli* K-12 J53-2 rif^r^ (*23*). PCR product sequences were then compared to reported ESBL sequences and assigned to specific types or clusters. To identify any epidemiologic relationship between the strains, they were compared by using enterobacterial repetitive intergenic consensus (ERIC)-PCR with ERIC1 and ERIC2 as primers (*24*,*25*). When strains had identical electrophoretic profiles with both ERIC1 and ERIC2 primers, they were considered identical, and only 1 isolate per electrophoretic profile type was selected for further analysis. Among the collection of 129 strains selected for the study, 86 strains were involved in infections (urinary tract infection [UTI]: 64, bacteriemia: 7, pus production from miscellaneous infections: 15), and 43 strains were isolated from colonization (rectal samples: 39, gastric aspirate: 1, abdominal drainage: 1, vaginal sample: 1, tracheal aspirate: 1) (Table 2). The collection included 55 strains that produced a TEM-type ESBL, 22 strains produced a SHV-type ESBL, and 52 strains produced a CTX-M type ESBL (Table 2).

**Table 1:** Sequence of primers used to detect *bla* genes*

PCR target	Primer name	Primer sequence	Reference or accession no.
*bla*_TEM_	A B	ATGAGTATTCAATTCCG CTGACAGTTACCAATGCTTA	(22)
*bla*_SVH_	P4 P5	GGTTATGCGTTATATTCGCC TTAGCGTTGCCAGTGCTC	(22)
*bla*_CTX-M_ (CTX-M-1 cluster)	MenA MenB	AAGACTGGGTGTGGCATTGA AGGCTGGGTGAAGTAAGTGA	X92506
*bla*_CTX-M_ (CTX-M-2 cluster)	M2A M2B	CTGGAAGCCCTGGAGAAAAG TACCTCGCTCCATTTATTGC	X92507
*bla*_CTX-M_ (CTX-M-9 cluster)	ToA ToB	GCTTTATGCGCAGACGAGTG GCCAGATCACCGCAATATCA	AF174129
*bla*_CTX-M_ (CTX-M-8 cluster)	A8 B8	GCCTGTATTTCGCTGTTG TGTCATTCGTCGTACCATAA	AF189721

*PCR, polymerase chain reaction.

**Table 2:** Distribution of ESBL types according to strain origin*

ESBL type (no. strains)	No. strains isolated from		
	UTI	Other infections	Colonization
TEM (55)			
TEM-24	11	9	11
TEM-52	3	0	7
TEM-21	5	1	2
TEM-3	1	0	3
TEM-10	0	0	1
TEM-20	1	0	0
SHV (22)			
SHV2	3	1	2
SHV4	1	1	1
SHV5	2	0	1
SHV12	5	4	1
CTX-M (52)			
CTX-M-1 cluster	20	2	7
CTX-M-2 cluster	3	0	1
CTX-M-9 cluster	9	4	6

*ESBL, extended-spectrum β-lactamase; UTI, urinary tract infection.

### Susceptibility Testing, Phylogenetic Grouping, and Virulence Factors

Susceptibility to ciprofloxacin was tested by the disk diffusion technique according to the guidelines of the Antibiogram Committee of the French Society for Microbiology (www.sfm.asso.fr) with MIC criteria of <1 mg/L (diameter >22 mm) used to define susceptibility. Phylogenetic grouping of the *E. coli* isolates was determined by a PCR-based method developed by Clermont et al. (*26*) that uses a combination of three DNA markers (*chuA*, *yjaA*, and an anonymous DNA fragment, TspE4.C2). Strains were assigned to phylogenetic groups on the basis of presence or absence of the three DNA fragments: *chuA*–, TspE4.C2–, group A; *chuA*–, Tspe4.C2+, group B1; *chuA*+, *yjaA*+, group B2; *chuA*+, *yjaA*–, group D. Because 2 possible profiles can be obtained for the groups A, B2, and D, each was subdivided as follows: *chuA*–, *yjaA*–, Tspe4.C2–, group A subgroup A_0_; *chuA*–, *yjaA*+, Tspe4.C2–, group A subgroup A_1_; *chuA*+, *yjaA*+, Tspe4.C2–, group B2 subgroup B2_2_; *chuA*+, *yjaA*+, Tspe4.C2+, group B2 subgroup B2_3_; *chuA*+, *yjaA*–, Tspe4.C2–, group D subgroup D_1_; *chuA*+, *yjaA*–, Tspe4.C2+, group D subgroup D_2_. Virulence genes (*pap*, *sfa*/*foc*, *hly*, *aer*) were detected from DNA by PCR as described previously (*15*,*27*). These genes code for 2 adhesins (pyelonephritis-associated pili system and S fimbrial adhesin), 1 toxin (α-hemolysin), and 1 iron captation system. These genes are good representatives of the intrinsic extraintestinal virulence of the strains (*28*).

### Statistical Analysis

Data were summarized in 2 two-way tables, and each table had 129 rows, one for each *E. coli* strain. The first table had 16 columns corresponding to the variables, origin of the strains, phylogenetic group or subgroup, type of ESBL, and virulence factors. The second table had 12 columns corresponding to the variables, phylogenetic groups, type of ESBL, and resistance to ciprofloxacin. For each column, each strain was coded as a binary code: present = 2, absent = 1. A factorial analysis of correspondence (FAC) (*29*) was conduced from this table with SPAD.N software (Cisia, Saint Mandé, France). To confirm the significance of the correlation observed with FAC, χ² tests were carried out.

## Results

### Characterization of ESBL Strains

Among the 129 *E. coli* strains analyzed, phylogenetic group B2, which is the source of most ExPEC clones, was represented by 36.4% of the strains (8.5% were subgroup B2_2_ and 27.9% were subgroup B2_3_). Phylogenetic group D, which is also a source of ExPEC but to a lesser extent, was represented by 25.5% of the strains (17% were subgroup D_1_ and 8.5% were subgroup D_2_). Of the remaining strains, phylogenetic groups A and B1 were represented by 27.9% (9.3% were subgroup A_0_ and 18.6% were subgroup A_1_) and 10% of the strains, respectively. The virulence determinants most represented in the collection were *aer* and *pap*, with 53 (41%) and 38 (29.5%) strains carrying these genes, respectively. Less prevalent were *sfa*/*foc* and *hly* determinants, with only 18 (14%) and 19 (15%) positive strains, respectively. Fluoroquinolone resistance was present in 34.8% of the strains.

ESBL-producing strains were found in all *E. coli* phylogenetic groups. Of the strains, 60% and 24% harbored at least 1 or 2 extraintestinal virulence determinants, respectively. Coresistance to fluoroquinolones was frequent.

### Multidimensional Analysis

To assess relationships between phylogenetic groups, VFs, type of ESBL produced, and origin of the strains (infection or colonization), a FAC was constructed with the 129 *E. coli* strains as individuals and the 16 characteristics as qualitative variables. Projections of the variables on the plane F1/F2 (Figure A), which accounted for 34.5% of the total variance, showed a correlation between the type of ESBL produced and several phylogenetic group/subgroups of *E. coli*. Thus, SHV type and subgroup B2_3_ are projected on the positive values of F1 and negative values of F2, whereas TEM type and subgroup B2_2_ are projected on the positive values of F1 and F2. CTX-M type and subgroup D_2_ are projected on the negative values of F1 and F2. Correlation between SHV type and subgroup B2_3_ was confirmed by χ² tests (p < 0.001) and the CTX-M type and the subgroup D_2_ (p < 0.001) (Table 3).

**Figure:**
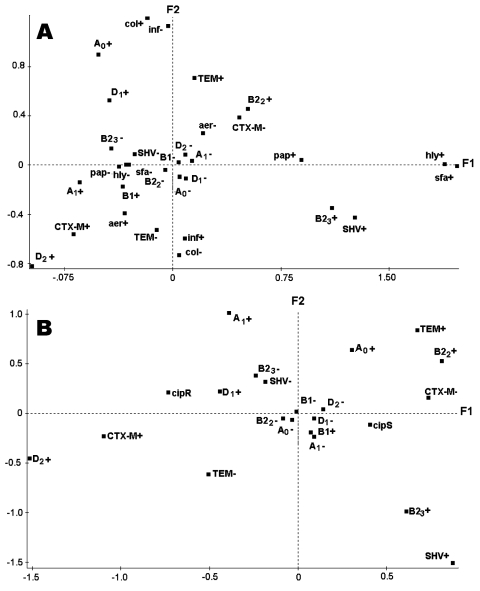
Graphic representation of the results of the factorial analysis of correspondence carried out with whole data from the 129 *Escherichia coli* strains. A) Projections of the variables on the F1/F2 plane: phylogenetic group and subgroups (A_0_, A_1_, B1, B2_2_, B2_3_, D_1_, and D_2_), type of extended-spectrum β-lactamase (ESBL) (TEM, SHV, CTX-M), virulence factors (pap, sfa, hly, aer), and the source infection (inf) or colonization (col). B) Projections of the variables on the F1/F2 plane: phylogenetic group and subgroups (A_0_, A_1_, B1, B2_2_, B2_3_, D_1_, and D_2_), type of ESBL (TEM, SHV, CTX-M), and ciprofloxacin resistance (cipR) or the ciprofloxacin susceptibility (cipS).

**Table 3:** Distribution of extended-spectrum β-lactamase types among *Escherichia coli* strains isolated from infection or colonization, according to phylogenetic group

Phylogenetic group	No. strains in the group (%)	No. (%) of isolates producing		
		TEM	SHV	CTX-M
Infection				
A_0_	5 (5.8)	2 (6.4)	1 (5.8)	2 (5.2)
A_1_	18 (20.9)	11 (35)	0	7 (18.4)
B1	9 (10.4)	2 (6.4)	3 (17.6)	4 (10.5)
B2_2_	7 (8.1)	5 (16)	1 (5.8)	1 (2.6)
B2_3_	28 (32.5)	8 (25.8)	10 (58.8)	10 (26.3)
D_1_	11 (12.7)	3 (9.6)	2 (11.7)	6 (15.7)
D_2_	8 (9.3)	0	0	8 (21)
All groups	86 (100)	31 (100)	17 (100)	38 (100)
Colonization				
A_0_	7 (16.2)	5 (20.8)	0	2 (14.2)
A_1_	6 (13.9)	2 (8.3)	0	4 (28)
B1	4 (9.3)	3 (12.5)	0	1 (7.1)
B2_2_	4 (9.3)	2 (8.3)	1 (20)	1 (7.1)
B2_3_	8 (18.6)	5 (20.8)	3 (60)	0
D_1_	11(25.5)	6 (25)	1 (20)	4 (28.4)
D_2_	3 (6.9)	1 (4.1)	0	2 (14.1)
All groups	43 (100)	24 (100)	5 (100)	14 (100)
All strains				
A_0_	12 (9.3)	7 (12.7)	1 (4.5)	4 (7.6)
A_1_	24 (18.6)	13 (23.6)	0	11 (21.1)
B1	13 (10)	5 (9)	3 (13.6)	5 (9.6)
B2_2_	11 (8.5)	7 (12.7)	2 (9)	2 (3.8)
B2_3_	36 (27.9)	13 (23.6)	13 (59)	10 (19.2)
D_1_	22 (17)	9 (16.3)	3 (13.6)	10 (19.2)
D_2_	11 (8.5)	1 (1.8)	0	10 (19.2)
All groups	129 (100)	55 (100)	22 (100)	52 (100)

As previously reported, *sfa*/*foc* and *hly* VFs were exclusively found in strains of the subgroups B2_2_ and B2_3_ (10,16). Pairwise comparisons between individual subgroups showed that subgroups B2_2_ and B2_3_ each had mean VF scores (1.45 and 1.8, respectively) significantly higher than either phylogenetic groups and subgroups A_0_, A_1_, B1, D_1_, or D_2_ (p < 0.02 for all comparisons), but they were not significantly different from one another. Likewise phylogenetic groups and subgroups A_0_, A_1_, B1, D_1_, and D_2_ were not significantly different from one another with respect to mean VF scores (mean scores 0.5, 0.66, 0.54, 0.5, and 0.63, respectively). When the type of ESBL produced was considered, the frequency of VFs was higher in SHV-producing strains (mean score = 1.8) than in TEM-producing strains (mean score = 0.96). The lowest frequency was found in the CTX-M-producing strains (mean score = 0.6). FAC stressed these 2 observations, as it showed that the *pap*, *sfa*/*foc*, and *hly* VFs were projected on the positive values of the first axis with the subgroup B2_3_ and the SHV type. The correlation between SHV type and the presence of the 3 VFs was also confirmed by χ² tests (*pap*, p < 0.01; *sfa*/*foc*, p < 0.001; *hly*, p < 0.001). Aerobactin was found in all the phylogenetic groups and subgroups, and no correlation was observed with the FAC (Table 4).

**Table 4:** Frequency of virulence factors among ciprofloxacin-susceptible and ciprofloxacin-resistant *Escherichia coli* strains involved in infection or colonization, according to extended-spectrum β-lactamase (ESBL) type

ESBL type (no. strains)	No. (%) strains carrying				Virulence factor mean score
	*pap*	*sfa*/*foc*	*hly*	*aer*	
Ciprofloxacin resistance					
TEM (15)	4 (20)	0	0	8 (53)	0.8
SHV (3)	0	0	0	1 (33)	0.33
CTX-M (27)	2 (7)	0	0	12 (44)	0.51
All types (45)	6 (13)	0	0	21 (46)	0.6
Ciprofloxacin sensitivity					
TEM (40)	14 (35)	9 (22)	9 22)	9 (22)	1
SHV (19)	13 (68)	8 (42)	9(47)	10(52)	2.1
CTX-M (25)	5 (20)	1 (4)	1 (4)	13 (52)	0.8
All types (84)	32 (55)	18 (21)	19 (22)	32 (38)	1.2
All strains					
TEM (55)	18 (33)	9 (16)	9 (16)	17 (31)	0.96
SHV (22)	13 (59)	8 (36)	9 (4)	11 (50)	1.8
CTX-M (52)	7 (13)	1 (2)	1 (2)	25 (48)	0.6
All types (129)	38 (29.5)	18 (14)	19 (15)	53 (41)	1

Projection of the colonization and infection variables on the plane showed that they were clearly distinguished by the first factor and that there was a correlation with some phylogenetic groups (Figure A). The colonization characteristic was projected on the positive values of F1 with phylogenetic subgroups A_0_ and D_1_. The association was close to significance (A_0_, p = 0.05; D_1_, p = 0.06): strains of subgroups A_0_ and D_1_ were isolated more frequently from colonization (relative risk [RR] of 3.15 and 2.34, respectively) (Table 3). If we consider the clones usually to be the major source of ExPEC, strains of the subgroup B2_2_ were equally distributed among the strains responsible for infection or colonization (8.1% versus 9.3%), but strains of subgroup B2_3_ were more numerous among the strains responsible for infection than for colonization (32.5% versus 18.6%); the correlation was close to significance (p = 0.09, RR = 2.11) (Table 3). TEM type was also projected on the positive values of F2 with the colonization characteristic, and the χ^2^ test confirmed the correlation (p = 0.03).

The mean VF score of the strains responsible for infection was significantly higher (p = 0.03) than the mean VF score of the strains responsible for colonization (1.1 and 0.76, respectively). However, when each VF was considered, only the frequency of aerobactin was significantly higher among the strains responsible for infection (p = 0.03) than the strains responsible for colonization.

To assess the relationships between phylogenetic groups and subgroups, ESBL type, and resistance to fluoroquinolones, a second FAC was performed, taking into account only these variables (Figure B). Projection of the variables on the plane F1/F2, which accounted for 34% of the total variance, showed a correlation between resistance to ciprofloxacin and type of ESBL produced. Thus, the ciprofloxacin-resistant characteristic was projected on the negative values of the first factor with CTX-M-type, and the ciprofloxacin-susceptible characteristic was projected on the positive values of the first factor with TEM and SHV types. Significant differences were observed between the rate of resistance to fluoroquinolones among the CTX-M- (51.9%) and among the SHV- and TEM-producing strains (13.6% and 27.7%, respectively): CTX-M type / SHV type, p = 0.002 and CTX-M type / TEM type, p = 0.009. FAC stressed also the correlation between the subgroup D_1_ and the resistance to ciprofloxacin, which were projected together on the negative values of the first factor and on the positive values of the second factor. The correlation was confirmed by the χ² test (p = 0.03). Strains of phylogenetic subgroup D_1_ had the highest resistance rate (54%), and strains of subgroups B2_2_, B2_3_, and A_0_ had the lowest resistance rates (18%, 25%, and 25%, respectively). Group/subgroups B1, D_2_, and A_1_ had ciprofloxacin resistance rates of 30.7%, 36%, and 45%, respectively. No significant difference was seen in the frequencies of ciprofloxacin resistance among strains from infection or colonization (38.3% versus 27.9%). The mean VF score of the ciprofloxacin-susceptible strains was significantly higher (p < 0.001) than the one of the ciprofloxacin-resistant strains (1.2 and 0.6, respectively) (Table 4). We found *hly* and *sfa*/*foc* exclusively in ciprofloxacin-susceptible strains, and the frequency of *pap* was significantly higher among ciprofloxacin-susceptible strains (p = 0.04) than among ciprofloxacin-resistant strains. No difference was observed in the frequency of aerobactin between the two groups (Table 4). Although the frequency of CTX-M type was higher among UTI strains than among non-UTI strains (Table 2), FAC analysis and χ² tests did not show any significant association between UTI strains, phylogenetic group or subgroup, individual VFs, and ciprofloxacin resistance (data not shown), which could explain some of the previously observed correlations.

Therefore, strains harboring ESBL of SHV and TEM types belonged preferentially to the B2 phylogenetic group. They possessed extraintestinal VFs, but ESBL TEM-type strains were more likely to be isolated from cases of colonization; they were also susceptible to fluoroquinolones. On the other hand, strains harboring ESBL of CTX-M type were associated with D_2_ phylogenetic subgroup, had few VFs, but were resistant to fluoroquinolones.

## Discussion

This study was designed to assess the role of the genetic background of strains of *E. coli* in the emergence of ESBL. Strains were sampled from hospitals in several distant areas, which allowed us to build up a collection of strains producing variants of the most prevalent ESBL types. Thus three groups of ESBL-types were collected, TEM-, SHV-, and CTX-M-type, having enough strains in each group to be compared. Spread of clones of ESBL-producing organisms can occur from cross-contamination among patients (*2*,*7*,*23*). Therefore, to avoid redundant strains, we used ERIC-PCR as a typing method, and strains with similar profiles were eliminated.

Several studies suggested that extraintestinal pathogenic *E. coli* strains are mostly derived from the B2 phylogenetic group and to a lesser extend from the D group (*15*,*16*,*30*–*34*). It had been estimated in collections dating from before the emergence of ESBL, or in collections not selected for ESBL production, that group B2 strains account for approximately two thirds of all extraintestinal *E. coli* infections, including UTI, bacteremia, meningitis, and other miscellaneous infections. When all ESBL-producing *E. coli* strains were considered, whatever their types were, group B2 represented only 39.4% of the strains responsible for infection in our study. Thus, production of ESBL among *E. coli* clinical strains isolated from infection was associated with shifts in phylogenetic distribution toward non-B2 phylogenetic groups, in particular groups D and A. The distribution of group B2 among strains isolated from infection or from colonization was not very different even if it was pointed out that subgroup B2_3_ strains had a tendency to be isolated more frequently in clinical infections. Johnson et al., in 1991 (*18*), observed that *E. coli* strains belonging to phylogenetic groups other than group B2 have a greater prevalence of antimicrobial resistance, such as to ampicillin, tetracycline, chloramphenicol, streptomycin, and sulfonamide; express significantly fewer virulence factors; and invade more commonly compromised hosts. ESBL-producing organisms, which are resistant to β-lactams, except carbapenems and cephamycins, and are often resistant to other antimicrobials, are responsible for nosocomial infections, mostly in immunocompromised patients. ESBL-producing organisms also frequently colonize the lower digestive tract, and therefore are a major source for ESBL propagation (*8*). This finding may explain why two thirds of the strains in our study were not traditional virulence clones of ExPEC but clones whose ability to cause infection is limited to compromised hosts, in whom antibiotic resistance might provide selective advantage.

ESBLs are acquired β-lactamases that are encoded mainly by genes located on plasmids (*2*). As such, they are a recent evolutionary development. Even if the genetic element that carries resistance is a mobile element, the multidimentional analysis showed a preferential association between the genetic background and the type of ESBL produced by the strains. Thus, an association was seen between SHV type and subgroup B2_3_, between TEM type and subgroup B2_2_, and CTX-M type and subgroup D_2_. Even more, the *pap*, *sfa*/*foc* and *hly* VFs were associated with the genotype SHV type/subgroup B2_3_, defining a potentially high-virulence group of ESBL-producing *E. coli* strains. In contrast, the genotype CTX-M type/subgroup D_2_, characterized by a low VF score, defined a potentially low-virulence group of ESBL-producing *E. coli* strains. The type of ESBL produced by *E. coli* could be a predictive factor for intrinsic virulence potential.

Organisms that produce ESBL are frequently resistant to other antimicrobial agents, such as aminoglycosides, tetracycline, and trimethoprim-sulfamethoxazole, as many of these additional resistance genes are encoded on the ESBL-associated plasmid. Fluoroquinolone resistance, which is also frequently associated with ESBL production, is usually chromosomally encoded, unlike the other coresistances. However, plasmid-mediated quinolone resistance has been discovered recently (*35*). Prevalence of fluoroquinolone resistance among ESBL-producing strains varies according to geographic regions (*36*), from 13.7% in Canada to 65.5% in the western Pacific. In our study, 34.8% of strains were resistant, which is close to the prevalence (34.2%) reported in Europe (*36*). Correlation with phylogenetic background and VF profiles showed highly fluoroquinolone-resistant strains of subgroup D_1_, with the lowest VF score and association with colonization. In contrast, strains of phylogenetic group B2, which had the highest VF score, were among the strains with the lowest fluoroquinolone-resistance rates. These data agree with the work of Johnson et al. (*19*,*37*) and show a clear trade-off between resistance to fluoroquinolones and virulence. In addition, our study highlights an association between these fluoroquinolone-resistant strains and CTX-M-producing strains, which are devoid of VFs. However, the search for the gene responsible for plasmid-mediated quinolone resistance, *qnr*, by PCR was negative in our collection of ESBL-producing strains (O. Zamfir, E. Denamur, C. Branger, unpub. data). Thus, the observed association is not due to a genetic link between resistance to expanded-spectrum β-lactams and quinolones on a mobile element, as was recently reported (*38*).

During the last 2 decades, most of the ESBL found in *E. coli* and, in general, in gram-negative bacilli, has been of TEM or SHV lineage. Recently TEM and SHV types have been replaced by CTX-M-type ESBL, whose emergence and proliferation are particularly noteworthy (*39*). The current spread may be explained in part by the ability of some insertion sequence elements to mobilize and promote the expression of β-lactamase (*40*). However, the high rate of fluoroquinolone resistance and the low virulence of the strains carrying CTX-M ESBL could provide them selective advantage to spread, especially under strong environmental antimicrobial pressure with fluoroquinolones.

In summary, mobile elements encoding ESBL are not randomly distributed among the genetic diversity of the *E. coli* species. The arrival, expression, and maintenance of such elements seem to be the result of complex interactions between the type of ESBL, the phylogenetic background, the intrinsic virulence of the strains, and the presence of associated fluoroquinolone resistance. Such complexity reflects very likely the diversity of ecologic niches with different selective pressures.
